# The Red Flour Beetle as a Model for Bacterial Oral Infections

**DOI:** 10.1371/journal.pone.0064638

**Published:** 2013-05-30

**Authors:** Barbara Milutinović, Clemens Stolpe, Robert Peuβ, Sophie A. O. Armitage, Joachim Kurtz

**Affiliations:** Institute for Evolution and Biodiversity, University of Münster, Münster, Germany; U. Kentucky, United States of America

## Abstract

Experimental infection systems are important for studying antagonistic interactions and coevolution between hosts and their pathogens. The red flour beetle *Tribolium castaneum* and the spore-forming bacterial insect pathogen *Bacillus thuringiensis* (*Bt*) are widely used and tractable model organisms. However, they have not been employed yet as an efficient experimental system to study host-pathogen interactions. We used a high throughput oral infection protocol to infect *T*. *castaneum* insects with coleopteran specific *B. thuringiensis* bv. *tenebrionis* (*Btt*) bacteria. We found that larval mortality depends on the dietary spore concentration and on the duration of exposure to the spores. Furthermore, differential susceptibility of larvae from different *T. castaneum* populations indicates that the host genetic background influences infection success. The recovery of high numbers of infectious spores from the cadavers indicates successful replication of bacteria in the host and suggests that *Btt* could establish infectious cycles in *T. castaneum* in nature. We were able to transfer plasmids from *Btt* to a non-pathogenic but genetically well-characterised *Bt* strain, which was thereafter able to successfully infect *T. castaneum*, suggesting that factors residing on the plasmids are important for the virulence of *Btt.* The availability of a genetically accessible strain will provide an ideal model for more in-depth analyses of pathogenicity factors during oral infections. Combined with the availability of the full genome sequence of *T. castaneum*, this system will enable analyses of host responses during infection, as well as addressing basic questions concerning host-parasite coevolution.

## Introduction

Insects are important model organisms for studying the evolution and mechanisms of immunity and host-pathogen interactions [1]–[4]. For example, experimental approaches have been established for oral inoculation of natural bacterial pathogens for the main insect model, the fruit fly *Drosophila melanogaster* [5]–[7], thereby adding a vital tool to the methodological repertoire of insect immunology. This has enabled the successful in-depth study of the pathology of bacterial infections [8], [9].

The red flour beetle *Tribolium castaneum* (Herbst 1797) has developed into a fully-fledged insect model organism [10]. The value of *T. castaneum* as an alternative insect model lies in the fact that, as a coleopteran, it shows a number of distinct differences to the fly and since it is evolutionarily more basal, it can be regarded as being more representative of other insects [11]–[13]. The availability of an expanding genetic and genomic toolbox that includes well-functioning systemic RNAi [14], [13] has made *T. castaneum* an upcoming model for a number of research fields [14], [11], [10], [13], including immunity and host-parasite interactions [15]–[17]. Furthermore, *T. castaneum* is a serious pest species in many areas of the world, leading to substantial losses in the nutritional value of stored agricultural products [18]. Therefore, there is a strong interest in research on pest management for this species.

*Bacillus thuringiensis* Berliner 1915 (*Bt*) is a Gram-positive bacterium that forms highly resistant endospores when nutrients in the environment become limiting. One of its main characteristics is that it produces plasmid-encoded crystalline inclusions (Cry proteins) during the sporulation phase, which are toxic to specific insect orders upon ingestion [19], [20]. The nomenclature of Cry toxins is based on amino acid identity [21]. Cry3 toxins are active towards some coleopterans and cross-order activity has been reported for some of the lepidopteran-specific Cry toxins (reviewed in [22]). The vast majority of studies have focussed on the toxicity of Cry toxins [23], [24], and several mechanisms for its mode of action have been proposed (reviewed in [25]). However, many insects, including *T. castaneum* have been shown to be refractory to purified toxins [26], [27] and mortality is observed only when bacterial spores are added to the diet [28]. The ingestion of spores and the following infection process that takes place in the gut and subsequently the haemolymph is considered a natural infection route for *Bt* [29]. Investigations on how the bacteria behave inside the host after infection and processes that act in addition to the toxins are therefore highly interesting from the viewpoint of host-parasite coevolution.

We exposed *T. castaneum* to *Btt* bacteria via oral route, and moreover made use of a genetically well characterised *Bt* strain. Since both the host and the pathogen are accessible to genetic manipulation, the system will enable detailed genetic analyses of the infection process and host-pathogen interactions. Importantly, *Bt* itself is an organism of utmost importance for basic and applied sciences [30]–[33]. Currently studied natural insect hosts of *B. thuringiensis* are mostly lepidopterans, such as the diamondback moth (*Plutella xylostella*) [34], the tobacco hornworm (*Manduca sexta*) [35], and the cotton bollworm (*Helicoverpa armigera*) [36] for which the full repertoire of genetic and genomic tools is not yet available. Likewise, even though *D. melanogaster* has been shown to die from exposure to *Bacillus* species, including *B. thuringiensis* [37], to our knowledge it has not been established as an experimental host for *Bt*. Transgenic *D. melanogaster* carrying lepidopteran (*M. sexta*) Cry receptor have been shown to become susceptible to *Bt* [38], suggesting a role for this specific receptor. However, such a system would not allow addressing the natural infection process or the genetic variation in the full range of factors that are relevant for susceptibility to *B. thuringiensis*.

Our first objective was to verify the most suitable bacterial strain for the investigation of this host-pathogen interaction. We identified *Bt morrisoni* bv. *tenebrionis* (*Btt*) as infective to *T. castaneum*, and further characterised the susceptibility of geographically diverse populations of *T. castaneum* to this strain. We then investigated the behaviour of the bacteria in the host and the time course of the infection. We also demonstrate the transfer of plasmids from *Btt* to a non-pathogenic but genetically characterised *Bt* strain, which thereby became able to successfully infect *T. castaneum*. The availability of such a genetically accessible strain will be most useful for a more in-depth analysis of this interaction in the future. The *T. castaneum – Bt* system proposed here shows the potential for in-depth experimental analyses of a coleopteran insect model host's interaction with this important pathogen.

## Results

### Insecticidal Activity of Different *Bt* Strains to *T. castaneum* Larvae

**Figure 1:**
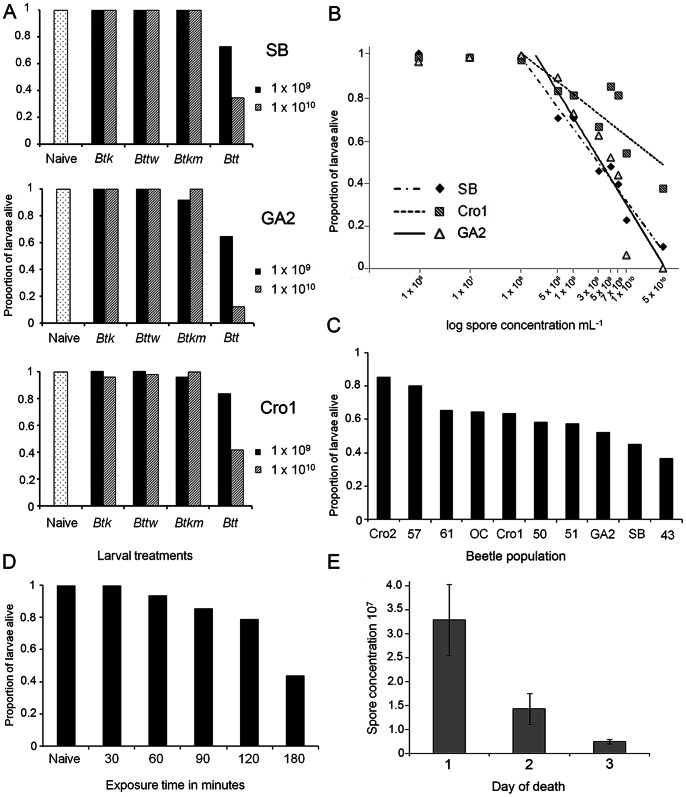
Infection of *T.* *castaneum* with *B. thuringiensis*. (A) Insecticidal activity of different *Bt* strains to *T. castaneum* larvae. Larval survival at day seven after constant exposure to flour containing *Bt* spores with two different concentrations, 10^9 ^mL^−1^ and 10^10^ mL^−1^ of four different *Bt* strains. Insect populations infected: San Bernardino (SB), Georgia 2 (GA-2), Croatia 1 (Cro1). (**B**) **Dose response curves for** ***Btt*** **infection.** Survival of three populations of *T. castaneum* larvae (SB, GA-2 and Cro1) at day seven after constant exposure to different concentrations of *Btt* spores in flour. We fitted linear regression curves to the log transformed values of spore concentrations, excluding the first two values where no mortality was induced (SB: survival = 3.68–0.15*log spore concentration, r^2^ = 0.97, p = <0.0001; Cro1: survival = 2.56–0.08*log spore concentration, r^2^ = 0.70, p<0.01; GA-2: survival = 4.35–0.18*log spore concentration, r^2^ = 0.89, p<0.001). (**C**) **Differences in susceptibility to** ***Btt*** **among ten beetle populations.** Survival of ten populations of *T. castaneum* larvae at day seven after constant exposure to *Btt* spores in flour (5×10^9^ mL^−1^). (**D**) **Limited exposure time to** ***Btt*** **spore-containing diet.** Survival of *T. castaneum* larvae (SB population) 24 hours after limited exposure time to spore-containing diet. Survival is shown for 24 hours post initial exposure (PIE) since 48 hours PIE no additional mortality was observed. (**E**) **Spore load of cadavers after infection with** ***Btt*** **.** Total spore number recovered from larvae that were collected on first, second and the third day of death.

We analysed the infectivity of four different *Bt* strains (Table 1) towards three different *T. castaneum* populations, the laboratory populations San Bernardino (SB) and Georgia 2 (GA-2) and the recently wild-collected Croatia 1 (Cro1) population (Figure 1A). When comparing the survival of the naïve group to the other treatments, only the *Btt* strain was able to induce significant mortality of *T. castaneum* larvae from all beetle populations. All other bacterial strains induced no significant mortality above the background level of the control insects (Figure 1A, Table S1). Larvae were kept constantly on the spore-containing diet (flour discs with spores in a 96 well plate), but the majority died within the first 24 hours after the exposure had started, with low mortality during the following days (Figure S1). Mortality was dependent on the spore concentration used to prepare the diet (1×10^9 ^mL^−1^: z = 4.463, p = ***<***0.0001, 1×10^10^ mL^−1^: z = 6.870, p = <0.0001), and SB and GA-2 population differed in their responses to the dietary spores (z = 2.484, p = 0.013, Table S1). Note that the total spore number that each larva was confronted with was approximately 4×10^7^ for the 1×10^9 ^mL^−1^ and 4×10^8^ for the 1×10^10^ mL^−1^ concentration of the original suspension used to prepare the diet (see materials and methods for details).

**Table 1:** *Bt* strains used to test their insecticidal activity to *T. castaneum* larvae.

*Bt* strain	BGSC Code	Cry toxin
*Bt morrisoni* bv. *tenebrionis (Btt)*	4AA1	3Aa
*Bt tolworthi (Bttw)*	4L3	3Ba
*Bt kumamotoensis (Btkm)*	4W1	3Bb
*Bt kurstaki (Btk)*	HD1	1Aa, 1Ab,1Ac, 2A, 2B

### Dose Response Curves for *Btt* Infection

The infection system allows for exposure to precise doses of dietary bacterial spores by adding different concentrations of spores per mL to the flour the experimental animals are kept on. This enabled us to study in more detail how the infection success of *Bt* depends on the spore exposure dose. For this, we used the *Btt* strain since it was the only strain causing significant mortality of *T. castaneum* larvae, and we used SB, GA-2 and Cro1 insect populations to test whether dose-response curves are population specific. For spore concentrations above a threshold concentration of 10^8^ spores per mL, all three populations showed a clear dose-dependent mortality, but the populations differed in the dietary concentration of spores required to kill a certain proportion of larvae (Figure 1B). Over a broad range of spore concentrations, the wild population Cro1 was found to be around 30–40% less susceptible than the two laboratory populations (Table S2). The lowest of the tested spore concentrations that resulted in reduced survival of larvae in all three populations was 5×10^8^ mL^−1^ (z = 3.643, p = 0.0003). When fed on the highest spore concentration tested (5×10^10^ mL^−1^), some larvae of the laboratory populations SB and GA-2 were still alive at day seven, but all had died by day 13 (data not shown).

### Differences in Susceptibility to *Btt* among ten Beetle Populations

Data obtained from the previous two experiments indicated that beetle populations may differ in their susceptibility to *Btt*. We therefore further compared the susceptibility of ten beetle populations (Table 2) to test this finding in more depth. Our ten populations showed substantial differences in their susceptibility to *Btt*, varying from 40%–85% survival after seven days of constant exposure to spores (Figure 1C). When compared to the standard laboratory population (SB), populations Cro1, Cro2, 50, 57, and 61 (Cro1: z = −2.527, p = 0.011, Cro2: z = −5.696, p = <0.0001, 50: z = −1.948, p = <0.0001, 57: z = −5.005, p = <0.003, 61: z = −3.004, p = 0.004) had higher survival rate when fed on *Btt* spore-containing diet (5×10^9^ mL^−1^), Table S3. The majority of larvae died during the first day of exposure; mortality was strongly reduced on the second day, and on the third day only a small percentage of the larvae died. In most of the populations, no mortality was recorded thereafter.

**Table 2:** *Tribolium castaneum* populations that were used in the study.

Beetle population	Year collected or established	Origin
Cro1	2010	Croatia
Cro2	2010	Croatia
SB	Unknown	California, USA
GA-2	1982	Georgia, USA
43	1988	Kyushu Island, Japan
50	2005	Indiana, USA
51	2006	Missouri, USA
57	2002	Peru
61	1996	Banos, Ecuador
OC Münster	2008	Outcrossed

### Adult Susceptibility to *Btt* and *Btk* Strains

Despite our observation that the adults (SB, GA-2 and Cro1 population) fed on the *Btt* spore-containing diet (5×10^9^ ml^−1^), no mortality was recorded during seven days of exposure. This experiment was repeated twice with the same results. It has previously been shown that adult *T. castaneum* are susceptible to purified toxin formulations of *Bt kurstaki* (*Btk*) [39]. We therefore tested the susceptibility of adults of the SB beetle population to *Btk* spores (5×10^9^ mL^−1^), however, similarly to *Btt*, no mortality was observed.

### Plasmid Exchange between *Btt* and the Non-pathogenic *Bt* 407*gfpcry* ^−^

**Figure 2:**
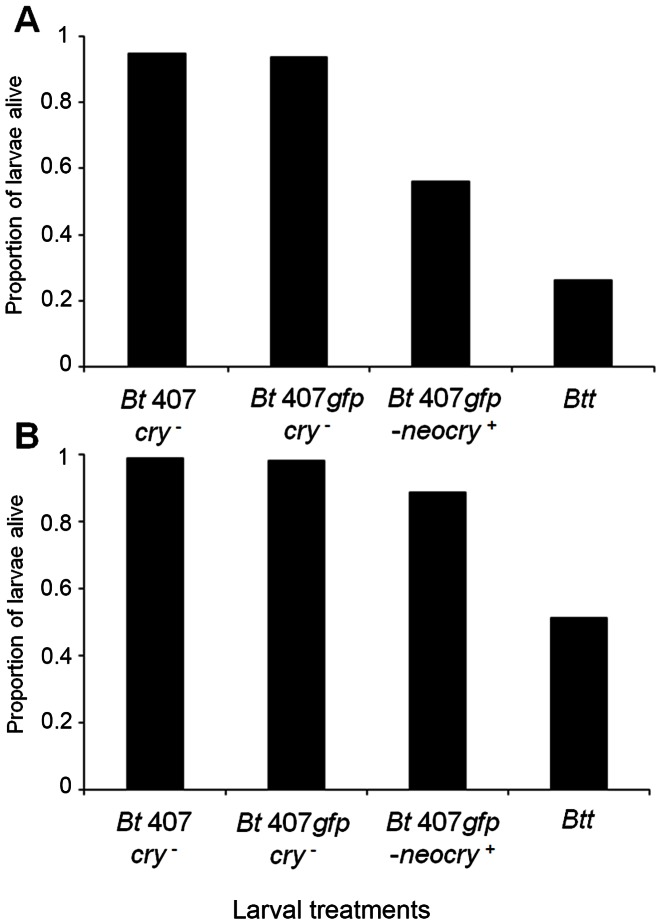
Pathogenicity of the conjugated *Bt* 407*gfp-neocry* ^+^strain. Survival of *T. castaneum* larvae at day seven after constant exposure to spores in flour of conjugated *Bt* 407*gfp-neocry* ^+^strain, *Btt* and the control strains (Table 3). Insect populations infected: A - San Bernardino (SB), B – Croatia 1 (Cro1). Spore concentration in flour: 5×10^9^ mL^−1^.

We were able to transfer pathogenicity factors from *Btt* (naturally neomycin resistant) to the non-pathogenic, green fluorescent protein (GFP)-expressing *Bt* 407*gfpcry* ^−^ that is erythromycin resistant (Table 3). After conjugation and selection on neomycin and erythromycin, we identified a number of double resistant clones. The selected clones were all of the 407 genetic background, which was confirmed by Rep-PCR (Figure S2) and which would imply that the *gfp* carrying plasmid was not transmittable from *Bt* 407*gfpcry* ^−^ to *Btt*. We tested for the presence of the *cry3A* gene with a *cry3A*-specific PCR (Figure S2). *Btt* carries two plasmids, a smaller one with unknown virulence factors and a large *cry*-carrying plasmid [40]. A large proportion (around 90%) of the tested clones was *cry* negative. Since the negative clones were able to grow on neomycin, this indicated that the resistance for this antibiotic may be present on the smaller plasmid. These clones were denoted as *Bt* 407*gfp*-*neocry* ^−^. We were not able to test the toxicity of these clones, since the bacteria did not sporulate in the presence of both antibiotics, even after two weeks of growth in spore-culturing conditions. Of the double resistant clones, five tested positive for *cry3A* and were denoted as *Bt* 407*gfp-neocry* ^+^. One clone was chosen for further analyses. This conjugated strain *Bt* 407*gfp-neocry* ^+^that had the large *cry*-carrying plasmid was able to induce considerable mortality in SB and Cro1 beetles (Figure 2). Mortality was lower compared to the original *Btt* strain, Table S4, Table S5). The mortality pattern during the seven days of spore exposure was similar to the *Btt* strain, with the majority of larvae dying on the first day.

**Table 3:** Plasmid exchange between *Btt* and the non-pathogenic *Bt* 407*gfpcry.*

*Bt* strain	Antibiotic resistance
*Bt morrisoni* bv. *tenebrionis* (*Btt*)	neo^R^
*Bt thuringiensis* Cry ^−^ (*Bt* 407*gfpcry* ^−^)	ery^R^
*Bt thuringiensis* Cry^+^(*Bt* 407*gfp-neocry* ^+^)	ery^R^, neo^R^
*Bt thuringiensis* (*Bt* 407*cry* ^−^)	–

We noticed that the large *cry*-carrying plasmid was rather stably retained in the conjugated *Bt* 407*gfp-neocry* ^+^strain. In the majority of cases where the conjugated strain was raised in the absence of antibiotics, the plasmid remained present. However, upon repeated freezing and thawing of glycerol stocks, the plasmid was lost at a higher rate. We observed plasmid loss also when the strain was raised with erythromycin alone, or with both antibiotics (erythromycin and neomycin) together. However, when raised with neomycin alone, the *cry* gene was retained (as detected by PCR), but the GFP signal was lost, suggesting that harbouring all three plasmids comes with a cost for the cells.

### Limited Exposure Time to *Btt* Spore-containing Diet

In the previous experiments, larvae were continuously kept on spore-containing flour. However, since most larvae died on the first day of exposure, continuous exposure may not be necessary to achieve mortality. Therefore, to analyse in more detail the behaviour of the ingested pathogen and the infection dynamics in the host, we limited the exposure time to the spore-containing diet. We therefore tested the exposure time necessary to induce mortality, and kept *T. castaneum* larvae (SB population) on spore-containing flour (*Btt*, 5x10^9^ spores ml^−1^) for between 30 and 180 minutes before transferring them to spore-free diet and followed their survival. Larval mortality 24 hours post initial exposure (PIE, here defined as the start of the 180 min. exposure period) occurred with only 60 minutes of exposure, although it was significantly different from the control treatment after 120 minutes (z = 2.311, p = 0.021, Table S6). After 180 minutes of exposure, mortality reached values equivalent to continuous exposure (Figure 1D) and no mortality was recorded 48 hours PIE. This suggests that the number of spores required to induce mortality is possibly rather low and that the first physiological changes in both the host and the parasite that contribute to mortality probably occur quite early in the process of infection.

### Larval Mortality Rate

**Figure 3:**
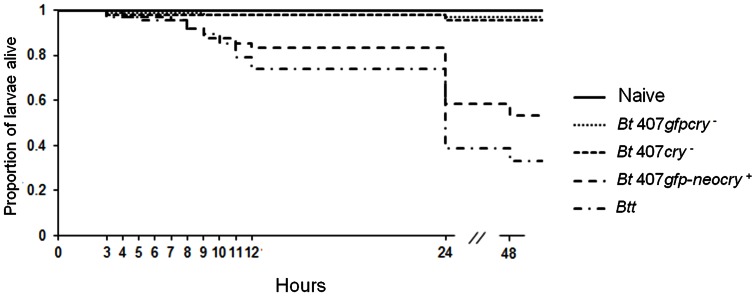
Larval mortality rate. Larvae of SB beetle population exposed for 3 hours to spore-containing diet. Mortality rate was monitored hourly starting from the third hour post initial exposure (PIE) till the twelfth hour PIE and then 24 and 48 hours PIE.

We used the information from this experiment to obtain a more complete picture of the course of larval mortality following the infection. We exposed larvae of the SB population for 180 minutes to 5x10^9^ spores ml ^−1^ (*Btt*, *Bt* 407*gfp-neocry* ^+^and the control strains, Table 3) and then transferred them to fresh flour without spores. We subsequently screened survival every hour until twelve hours PIE, and then again at 24h and 48 hours PIE (Figure 3). A small number of larvae (4%) had already died during the 180 min. of exposure. The survival curves for both pathogenic strains (*Btt* and *Bt* 407*gfp-neocry* ^+^) followed the same mortality trend. Larvae started dying seven to eight hours PIE and mortality was more strongly induced at 10 and 12 hours PIE. However, most larvae died between 12 and 24 hours PIE. Although the survival curves of the pathogenic strains showed similar mortality rates, *Bt* 407*gfp-neocry* ^+^again induced lower mortality in comparison to *Btt* (z = 5.164, p = 0.007, Figure 3, Table S7).

### Infection Dynamics of *Btt* Infection

To describe the infection process in more detail, we monitored bacterial growth in the host (SB beetle population) hourly until 13 hours PIE, since *Btt* induced fast mortality in previous experiments. Larvae were exposed for a maximum of three hours to *Btt* spores (5x10^9^ spores ml^−1^), and observations started at two hours PIE. At two hours PIE, only germinating spores, which appeared dark under phase contrast, were observed in the midgut. After about two to three hours PIE, the spores started to elongate into growing cells. Already at four to six hours PIE, 23% of infected larval midguts had a high load of vegetative cells, rising to 51% of the larvae seven to nine hours PIE. The midgut was entirely filled with bacteria, which seemed to be retained inside the midgut since they were not entering the surrounding buffer through the midgut wall after dissection. We were not able to observe bacteria in the haemolymph at any time point, although the haemolymph appeared darker in colour in some individuals suggesting the activation of an immune response (phenoloxidase reaction). After the originally ingested spores had germinated, only vegetative cells were observed during the following time-points. The formation of new spores was only observed in larvae that had been dead for one to two days, and after seven days the vast majority of bacteria inside the larvae had sporulated. Overall, in about 30% of the cases, neither germinated spores nor vegetative cells could be observed in the midgut.

### Spore Load of Cadavers after Infection with *Btt*

We measured the spore load of larval cadavers that had died from *Btt* infection. The mean total spore number per larvae was 1.83×10^7^, but it varied considerably among cadavers. We compared the spore load of larvae that had died on different days after being constantly exposed to the dietary spores (Figure 1E). The spore load was significantly higher in larvae that had died on the first day as compared to those that had died on the third day after spore exposure (Wilcoxon test, χ^2^ = 9.06, df = 2, p = 0.011, Figure 1E, error bars 1+/− SE). The spore load recovered from cadavers was much higher than the amount of spores larvae had originally ingested (see materials and methods), which is indicative of successful replication of *Btt* inside *T. castaneum*.

## Discussion

The red flour beetle *T. castaneum* and the bacterium *B. thuringiensis (Bt)* provide a useful oral infection model system for experimental studies of host-pathogen interactions. The system enables the simultaneous study of bacterial infection strategies and responses of the host in a well-studied insect model organism.

The four *Bt* strains that we tested carry different Cry toxins (Table 1) and have previously been shown to be able to induce mortality in coleopterans. We found that only the Cry3Aa producing strain, *Btt*, resulted in significant mortality of *T. castaneum* larvae when exposed to spore - toxin mixtures. The main factor causing pathogenesis of the *Btt* strain is not fully clear. *Btt* produces the Cry3Aa toxin, which when applied in its purified form (without the addition of spores), caused mortality to the yellow mealworm beetle *Tenebrio molitor* but not to *T. castaneum* [27]. A recent study [41] reported a very low mortality in *T. castaneum* when exposed to spore-crystal mixtures of Cry3Aa producing strain. The different mortality observed in this study might come from a different bacterial chromosomal background or from differences in spore concentrations that were provided in the diet. Heimpel and Angus (1960) [26] categorised *Bt* susceptible lepidopteran insects into three types. The first and second types are susceptible when they are subjected to the toxin preparations alone, with differences in the speed with which mortality is induced. The third type of insects are not susceptible to the toxin alone, but a spore-toxin formulation is required for pathogenesis. Since the purified toxins from the *Btt* isolate are not sufficient to induce mortality in *T. castaneum* larvae, infection success here as well might rely on spore - crystal synergism, as suggested previously by Li et al [28]. Further research is necessary to determine the exact role of the Cry protein and the possible relevance of spore - crystal interactions in the infection process in this system.

*Btt* was originally isolated from a larva of *T. molitor* [42], a species which is closely related to *T. castaneum*. The nucleotide sequence of *Btt*’s *cry* gene [43], [44] is identical or very similar to the toxin genes of other isolates that have been found to induce mortality of coleopterans [45]–[47]. *T. castaneum* larvae were shown to be susceptible to *Bt* isolates from Egypt [48] and to strains isolated in Pakistan [49], but no further information on these isolates is available. To the best of our knowledge, the present study is the first showing *T. castaneum* susceptibility to the *Btt* strain. In contrast to oral infections, studies where *Bt* has been introduced into the haemocoel via septic wounding, non-coleopteran *Bt* strains were able to induce significant mortality [50], [51]. Such septic infection, which may also occur in nature, circumvents the infection processes in the gut, where specificity is mediated through Cry proteins, which bind specifically to host receptors in the gut epithelium [20], [52].

We were able to transfer pathogenicity from *Btt* to a non-coleopteran strain of *Bt* (*Bt* 407*gfpcry* ^−^) through the transfer of plasmids. Mortality induced by the conjugated strain was somewhat lower than with *Btt*, suggesting that additional virulence factors might reside on the chromosome of the original pathogen. Alternatively, if the Cry protein plays a crucial role in acting synergistically with the spores to cause mortality, the lower virulence might be caused by a lower copy number of the transferred plasmid or reduced expression of the *cry* toxin gene in the conjugated strain. The exchange of plasmids from *Btt* to a non-pathogenic strain may also be interesting to assess the role of the exchange of genetic material for the evolution of pathogens and as a factor contributing to the maintenance of genetic diversity and virulence in natural *Bacillus* populations. Plasmids can easily be exchanged in some *Bt* strains, which may broaden the host range of these bacteria.

Most parts of the gut of *T. castaneum* are far less basic than typical lepidopteran guts [53], such that spores probably start to germinate immediately after ingestion, as they do in vitro [54]. For this reason, infection can potentially depend on the early toxin-induced damage and reduced gut peristalsis, which enables the bacteria population to grow and remain in the midgut, but this needs to be experimentally verified. Note that food passage from the mouth to the ileum is only 60 minutes in *T. castaneum* [53]. We observed rapid bacteria proliferation in the gut only a few hours after the feeding had started, which was not observed within the spore and flour/yeast mixture, so this cannot be attributed to the bacteria feeding on the beetle diet.

It would be interesting to investigate in more detail the reasons for differential susceptibility to *Btt* of larvae from different populations (Figure 1C). Such differences may be due to a number of reasons, including differences in the immune responses of the different populations against the bacteria. Resistance may be related to genetic diversity of the host, since both recently captured populations and the outcrossed line (OC) showed rather high resistance, compared to most of the laboratory lines. Alternatively, populations may differ in their associated microbiota, which might play a role, even though the midgut microbiota is not the sole reason for the infection success of *Bt* [55], [29].

Adults seemed to be resistant when subjected to the same dose of spore-crystal preparations as larvae (5×10^9^ spores ml^−1^). By examining the spore discs, we observed that the adults did not avoid the infectious diet. The potential reasons for adult resistance may include superior processing of the toxin in the midgut [56], but also immune responses that are more efficient against *Bt*. Moreover, there are morphological differences between the midgut of larvae and adults, with adults having numerous regenerative crypts along the surface of the midgut [57], [58]. This could potentially confer resistance through faster regeneration of epithelial cells as suggested by Ferre and van Rie [59], but this hypothesis needs further investigation. Interestingly, previous studies showed that commercial formulations of purified toxins from a lepidopteran specific strain *Bt kurstaki*, induced mortality in *T. castaneum* adults [39]. However in our study, spore - toxin preparations of *Btk* induced mortality neither in adults nor in larvae.

Most of the *Bt* infection scenarios have been described for lepidopteran insects [60]–[63] whose midgut physiology markedly differs from coleopteran insects. *Bt* has been reported to cause death through general septicaemia [64]–[66], [29] by invading host tissues from the midgut. This may involve repeated resporulation of the vegetative cells in the midgut, which facilitates the production of highly concentrated Cry crystals [67], [68], [66]. The course of *Bt* infection in *T. castaneum* seems to differ from the infection process described for lepidopteran hosts, and does not follow the expectations for a typical intoxication process as observed in other insects. We could not detect bacteria in the hemolymph of live larvae, nor did we observe the formation of new spores as long as the insect was alive, although it is possible that numbers of bacteria in the haemolymph or spores in the gut could have been below our detection limit. A possible reason as to why resporulation may not be required is that vegetative cells also express the *cry* gene in the *Btt* strain [69].

The infection process in the *T. castaneum* larval gut was fast and mortality was rapidly induced. Fast killing may be advantageous for *Btt* since as long as the host is alive it has to overcome its immune system. By killing quickly, *Btt* can exploit the hosts' nutrients and sporulate, which enables further infections and pathogen propagation. This strategy may explain our somewhat puzzling observation that the pathogen achieved lower spore load in larvae that died later than those that died on the first day after exposure, which differs from the observations made in lepidopteran insects [70] where insects that died on the third day had more spores than those that died earlier or later after infection. Different host species were shown to offer a more favourable environment for *Bt* replication than the others [71], a phenomenon which is present within one species and is time-of death dependant; nevertheless, it is different for different *Bt* species that might have evolved specialisations for different insect orders.

The ecology of *Bt* is not completely understood since most of the *Bt* spores are abundantly found where the target hosts are not always present [72]. Although transmission of *Bt* in nature is not well characterised, a higher prevalence of entomopathogenic (toxin-carrying) *Bacillus* isolates in soil was correlated with the presence of insect cadavers in a field trial, and specialisation of a certain isolate for lepidopteran insects has been suggested [72]. Of particular relevance to the ecology of *T. castaneum* is the observation that *Bt* has been isolated from animal food mills [73] and that *Bt* strains isolated from granaries have been shown to be able to induce mortality in *T. castaneum* [48]. Moreover, a *Bt* isolate that has a highly similar *cry* gene sequence to the sequence of the *cry3Aa* gene from the *tenebrionis* strain was isolated from dead *Tribolium* sp. [47]. The cannibalistic nature of *T. castaneum* and other *Tribolium* species [53] provides the opportunity for them to come into contact with a high dose of spores if they cannibalise larval cadavers. In support of this hypothesis, we observed that some larvae that were allowed to feed on three week old infected larval cadavers for two days, subsequently died and their bodies were loaded with *Btt* (data not shown). Although more detailed experiments are needed to verify this observation, it tentatively suggests that the reproductive cycle of *Bt* can be completed in *T. castaneum* in nature.

## Materials and Methods

### Insects, Bacteria and Infection Protocol

#### Insects

Our study is based on eight laboratory and two wild populations of *T. castaneum* (Table 2). Genetic differentiation between the populations and some degree of inbreeding might be expected in the laboratory populations due to potential genetic bottlenecks at the time of collection and the time for which they have been kept in the laboratory [74], [75]. The San Bernardino population (SB) originates from Alexander Sokoloff, California. The outcrossed population OC Münster was produced in our laboratory by crossing 10 different laboratory populations: 43, 50, 51, 52, 53, 55, 57, 58, 59, 61, which had been provided by Michael Wade (Indiana University, Bloomington, USA), together with the GA-2 population. The Croatia 1 (Cro1) and Croatia 2 (Cro2) populations are presumably the most genetically diverse since they were collected recently (Croatia, May 2010: Cro1∶45° 48′ 55.98′′, 16° 17′ 12.7968′′, Cro2∶46° 0′ 11.9628′′, 15° 50′ 39.195′′), and were established from multiple individuals from random mating pairs (165 pairs for Cro1 and 27 pairs for Cro2). The offspring of the pairs were used to establish the stock populations. Both of the wild populations are kept as large stock populations (ca. 10,000 individuals each) and were allowed to adapt to laboratory conditions for about 14 generations (1 year and 6 months) before the experiments started. All beetles were kept on heat-sterilised (75°C) organic white flour (type 550) with 5% brewer’s yeast at 30°C, 70% humidity and a 12h/12h light-dark cycle.

#### Bacterial strains

In this study, the susceptibility of *T. castaneum* to *Bt* bacterial strains was investigated in order to find the most suitable strain for investigation of host-pathogen interactions. Strains for the infections were chosen according to their Cry toxins (Table 1). *Bt tolworthi* and *Bt kumamotoensis* both carry toxins that are toxic against coleopteran insects [76], [77]. *Bt morrisoni* bv. *tenebrionis* is toxic to coleopterans [42], [78], [79]. *Bt kurstaki* is a lepidopteran-specific strain although purified toxins were found to be active against *T. castaneum* adults [39], [22]. All *B. thuringiensis* strains were provided by the *Bacillus* Genetic Stock Center (BGSC, Ohio State University, USA) except for the strains *Bt* 407*cry* ^−^ and *Bt* 407*gfpcry* ^−^ [80], the latter of which carries a green fluorescent protein (GFP) marker [81]. These strains were kindly provided by Dr. Christina Nielsen-Leroux, Institut National de Recherche Agronomique, La Minière, 78285 Guyancourt Cedex, France.

#### Production of spore-crystal preparations

Spores were freshly produced before each infection using a modified version of a previously described protocol [82]. Vegetative cells and spores were cultured at 30°C. Bacteria from a glycerol stock (stored at -80°C) were plated on LB agar and grown overnight. This was done freshly before each infection to prevent loss of pathogenicity by long-term storage of bacteria on LB agar plates. The following day, 5 ml of BT medium (w/V–0.75% bacto peptone (Sigma), 0.1% glucose, 0.34% KH_2_PO_4_, 0.435% K_2_HPO_4_) was inoculated with one bacterial colony with the addition of 25 µL of salt solution (w/V–2.46% MgSO_4_, 0.04% MnSO_4_, 0.28% ZnSO_4_, and 0.40% FeSO_4_) and 6.25 µL of 1M CaCl_2_×2H_2_O and allowed to grow overnight on a bacterial shaker at 200 rpm. The following day, the resulting bacteria suspension, 5 mL of salt solution and 250 µL of 1M CaCl_2_×2H_2_O were added to 1 L of BT medium, and it was further incubated for a total of seven days in darkness. On day four, another 5 mL of salt solution and 250 µL of 1M CaCl_2_×2H_2_O were added. After seven days the suspension was centrifuged at 4000 rpm for 15 minutes, washed once in phosphate buffered saline (PBS) and then resuspended in PBS. The spores were counted with a Thoma counting chamber. Such spore preparations together with their crystals (spore-crystal preparations) were stored for a maximum of three days at room temperature and protected from light until they were used in experiments.

#### General infection protocol

For the infection of *T. castaneum* larvae, a modified protocol from Oppert (2010) [83] was used. The desired spore concentrations were adjusted by adding PBS, and 0.15 g of heat-sterilized flour with yeast was added per ml of spore suspension. Forty microliters of the resulting liquid diet was pipetted into each well of a 96-well plate (Sarstedt, Germany) under sterile conditions. The diet for the control insects was made in the same way but without the addition of spores. The open 96-well plates were then placed in plastic boxes (Tupperware), three in one box. Six holes were punctured in the lids of boxes (3 cm diameter) and plugged with foam stoppers (K-TK e.K., Germany) (4.2 cm diameter) to allow the air to circulate. The boxes were placed in a 50°C oven overnight to allow the spore-crystal discs to dry. Once the spore-crystal suspension had been mixed with flour it was only used on that same day in order to prevent spore germination and bacteria growth in the medium. The drying process did not allow for any spore germination and bacterial proliferation in the disc, which was confirmed by examining the disc under the microscope (400×magnification) before the infection. After the drying process, one larvae was added per well and the 96-well plates were sealed with transparent self-adhesive tape and holes were punctured to allow air circulation in each well. The 96-well plates were placed back into the plastic boxes and were kept as described before at 30°C and 70% humidity for infection. This protocol minimises the risk of contamination with spores and is suitable for rapid infection of a large number of individuals. The larvae remain constantly visible, which enables easy screening of survival (up to 3000 individuals per person, per hour). For laboratory surface sterilisation, 4% Incidin Active (Ecolab) was used. For each infection, 13–14 day old larvae (approximately 4 mm long) descending from approximately 200–300 one month old parents were allowed to feed for varying amounts of time, depending on the experimental setup. Since the *Btt* spores were homogenously mixed into the flour, the larvae are unlikely to selectively avoid taking up the spores from their food. However, an avoidance strategy could be to stop feeding when food is recognised as infectious. *T. castaneum* larvae can tolerate starvation for a maximum of 2 weeks [53], such that it would be possible that the exposed larvae that did not die early on during the exposure stopped feeding and died from starvation later on. To exclude this possibility, we verified that larvae had fed during the days of exposure by examining the flour feeding discs. The majority of larvae had eaten; however the feeding rate seemed reduced in comparison to control animals. Adult beetles (approximately two weeks post eclosion) were infected in the same way. Dead larvae were recognisable by the black body colour, or their immobility when touched with the tip of an injection needle and the relaxation of their legs.

The concentration of spores used in the experiments is expressed as concentration of spores per mL of the original suspension that was used to prepare the diet. Since the liquid evaporates during the overnight drying process of the flour discs, spores per mL can be expressed as spores per 150 µg of flour with yeast, which was the amount of flour that was added per mL of suspension. Each larva was confronted with 40 µL of the spore-containing liquid diet, therefore the total spore number per disc is approximately the spore concentration per mL divided by 25. Furthermore, larvae eat a small portion of this diet, which would indicate that the number of spores necessary to cause mortality is potentially low.

### Experimental Design

#### Insecticidal activity of different *Bt* strains to *T. castaneum* larvae

We analysed the susceptibility of three beetle populations (SB, GA-2 and Cro1, Table 2) to four different *Bt* strains whose toxins or spore-toxin preparations have previously shown toxicity towards coleopteran insects: *Btt*, *Btk*, *Btkm*, *Bttw* (Table 1). Spore concentrations of 1×10^9^ and 1×10^10^ ml^−1^ were tested for each bacterial strain. Larvae were kept constantly on spore-containing diet and the survival was assessed daily for seven days. Forty eight larvae were used for each of the treatment and the control groups.

#### Dose response curves for *Btt* infection

To test the insecticidal activity of different spore concentrations of *Btt*, a dose response curve was performed using the following concentrations of spores per ml^−1^∶1×10^6^, 1×10^7^, 1×10^8^, 5×10^8^, 1×10^9^, 3×10^9^, 5×10^9^, 7×10^9^, 1×10^10^ and 5×10^10^. Larvae from the SB, GA-2 and Cro1 populations were kept constantly on spore-containing diet and survival was assessed daily for seven days and then on the 13^th^ day. Forty eight larvae were used for each of the treatment and the control groups.

#### Differences in susceptibility to *Btt* among ten beetle populations

To test the susceptibility of ten beetle populations that were collected from different regions of the world (Table 2), the *Btt* spore concentration was adjusted to 5×10^9^ ml^−1^. Larvae were kept constantly on spore-containing diet and the survival was measured daily for seven days. Ninety six larvae were used for each of the treatment and the control groups.

#### Adult susceptibility to the *Btt* and *Btk* strains

The susceptibility of adults (SB, GA-2 and Cro1) was tested with a *Btt* spore concentration of 5×10^9^ ml^−1^. In a previous study it was shown that *T. castaneum* adults are susceptible to purified toxins of the *Btk* strain [39], therefore in a separate experiment, we tested the susceptibility of beetles from the SB population to *Btk* spores (5×10^9^ ml^−1^). The beetles were kept constantly on spore-containing diet and survival was assesses daily for seven days. Forty eight adults were used for each of the treatments and for the control group.

#### Plasmid exchange between *Btt* and the non-pathogenic *Bt* 407*gfpcry ^–^*

The *Bt* 407*gfpcry* ^−^ strain [80] is cured of a large Cry-carrying plasmid and carries a GFP marker linked to erythromycin resistance (pHT315-*paphA3':gfp*, [81]). The strain is well genetically characterised and can be easily genetically manipulated [84]–[88]. Since the strain does not induce mortality in *T. castaneum,* we transferred plasmids via conjugation from the *Btt* strain in order to test whether we could also make it pathogenic. *Btt* carries two plasmids, a smaller one and a large plasmid that carries the *cry* gene together with other potential pathogenicity factors [40]. *Btt* is naturally neomycin resistant [89]. Bacterial conjugation was performed as described previously [90]. The donor and recipient strains were grown separately at 30°C, 200 rpm, in Luria Broth (LB) medium with appropriate antibiotics overnight and were subsequently diluted 1∶100 into 7 ml of pre-warmed LB medium. Cultures were grown to an optical density (OD 600) of 0.5, and 250 µl of each strain were mixed together and incubated at 30°C and 180 rpm for 3 hours. To select for transconjugants, the suspension was plated on LB agar plates with neomycin (15 µg/mL) and erythromycin (10 µg/mL) and grown overnight. Individual colonies were screened by colony PCR (1. 2′–94°C, 2. 20′′–94°C, 3. 20′′–57°C, 4. 40′′–72°C (2.–4.×35), 5. 3–72°C), using the primers Col1A and Col1B [91]. Before each experiment with the conjugated strain, the Cry3A gene was confirmed as present by heating 5 µl of spore suspension for 20 minutes at 90°C and the same PCR protocol as above was used with 2 µL of spore suspension. The genomic background of bacterial strains obtained after the conjugation was confirmed by repetitive extragenic palindromic sequence-based PCR analysis (Rep-PCR) as previously described [92], this is a DNA fingerprinting technique based on the generation of distinctive electrophoretic patterns via primers designed for Rep sequences.

#### Bioassay with the conjugated strain

The toxicity of the conjugated strain *Bt* 407*gfp-neocry* ^+^was tested on larvae from SB and Cro1 beetle populations using the general infection protocol as mentioned previously. Strains that were used in this bioassay are summarised in Table 3. Besides *Btt* and the newly created *Bt* 407*gfp-neocry* ^+^, *Bt* 407*cry* ^−^ and *Bt* 407*gfpcry* ^−^ were used to control for the presence of different plasmids. Larvae were kept constantly on the spore-containing diet for seven days and survival was assessed daily. A sample size of ninety six larvae was used for each of the treatments.

#### Limited exposure time to *Btt* spore-containing diet

As observed in vitro in LB medium, spores germinate and elongate into vegetative cells in about 2.5 hours. We therefore expected the earliest formation of vegetative cells in the midgut to start at about 2.5 hours after the start of the exposure time. To analyse the infection dynamics after exposure to the spore-containing diet (5×10^9^ spores ml ^−1^) we limited the exposure time, i.e. larvae of the SB beetle population were allowed to feed for 30, 60, 90, 120 and 180 minutes, after which time they were transferred to spore-free flour. Their survival was assessed daily for three days. Forty eight larvae were used for each of the treatment and the control group.

#### Larval mortality rate

Larval death rate was measured hourly to obtain a more detailed picture of the mortality dynamics. To test whether the *Btt* and the *Bt* 407*gfp-neocry* ^+^differ, both strains were used in this experiment together with the control *Bt* strains (Table 3). Larvae of the SB beetle population were exposed to spore-containing diet (5×10^9^ spores ml ^−1^) for three hours after which they were transferred to spore-free flour. Survival was assessed hourly until the twelfth hour post initial exposure (PIE). A sample size of 48 larvae was used for each of the treatment and the control groups.

#### Infection dynamics of *Btt* infection

To analyse infection dynamics, larvae of the SB beetle population were exposed to *Btt* spores (5×10^9^ spores ml ^−1^) for three hours after which they were transferred to spore-free flour. Larvae were collected hourly until the thirteenth hour PIE. To observe whether *Btt* bacteria are able to invade the haemolymph from the midgut, each larva was first punctured dorsally with the tip of an injection needle (0.3 mm diameter) between the first and the second segment and the haemolymph was collected with a 1 µL capillary (Hirschmann GmbH). The amount of haemolymph that could be collected was on average about 0.1 µL. In several cases the haemolymph extraction was unsuccessful because the infection had already progressed so far that the body had become soft. The haemolymph was added to a droplet of PBS buffer on a microscope slide and was observed under the microscope using phase contrast (400×magnification). The same larva was then ice anesthetized, placed on a Petri dish and the first and the last segment were removed with a razor blade. A drop of PBS was added and the gut was carefully pulled out with a pair of forceps. Bacteria were observed after the midgut was homogenised with a pair of needles (200×and 400×magnifications). In total, 120 larvae were used for the analysis. Because the alimentary canal isolation and the analysis itself were time consuming, the same time points were done across different days. The observed characteristics of infection dynamics were similar for the same time point when analysed on different days. On average twenty four larvae were analysed per time point.

#### Spore load of cadavers after infection with *Btt*

Larvae of the Cro1 population were kept constantly on spore-containing diet (*Btt*, 5×10^9^ spores ml ^−1^) and to quantify the spore load, larvae that died on the first, second and third day (n = 6 for each day) were separated daily. To ensure complete sporulation, cadavers were used that were ten days old. Cadavers were individually homogenised with a pestle in 200 µL of PBS. The suspension was subsequently pushed through a cell strainer with a 40 µm nylon mesh (BD Biosciences) by using a pipette. The spores were counted with a flow cytometer (BDFacsCanto II) using 4.5 µm green fluorescent beads (Polysciences) as a reference, and analysed using BD FACSDiva Software. To estimate the mean total spore number in cadavers, 56 larval cadavers were randomly picked after seven days of constant exposure of larvae to the spores (*Btt*, 5×10^9^ spores ml ^−1^).

### Statistical analyses

Survival experiments were analysed using the R statistical package (R Development Core Team, 2011) version 2.11.1. Within R we used the Cox proportional hazard model (‘survival’ library: Therneau [and original Splus-> R port by Lumley] 2011) to test the effect of the treatment on survival. In some cases the control or treatment groups had 100% survival. Because no event occurred, there was no contribution to the likelihood, and a cox model could not be fitted. Therefore we denoted one individual in the group as dead at the first timepoint, allowing us to fit the model. In the experiments testing different bacterial strains and concentrations, and for the dose response curve, we started with the full model (e.g., fullmodel <-coxph (Surv (timeofdeath, censor) ∼ Beetle population * Bacteria concentration) and then performed model simplification by backwards elimination of non-significant terms. The data for the spore load comparison and the regression analysis were analysed with JMP version 9 for Mac. The data for the comparison of cadaver spore load were not normally distributed (Shapiro-Wilk test) and did not have equal variances (Levene test). We therefore performed a nonparametric Kruskal-Wallis test to analyse the effect of treatment (day of death). Pairwise comparisons were then done for each of the three days in turn (Wilcoxon test) and to reduce the probability of type 1 errors we performed a Bonferroni correction (α = 0.0169).

## Supporting Information

Figure S1 **Insecticidal activity of different** ***Bt*** **strains to** ***T. castaneum*** **larvae - survival during the seven days of exposure.** Larval survival during the seven days of constant exposure to flour containing *Bt* spores with two different concentrations, 10^9 ^mL^−1^ and 10^10^ mL^−1^ of four different *Bt* strains. Insect populations infected: A - San Bernardino (SB), B - Georgia 2 (GA-2), C - Croatia 1 (Cro1).
(TIF)

Figure S2 **Characterisation of bacterial clones after the conjugation.** A - *Bt* vegetative cells, phase contrast merged with fluorescence (GFP) microscopy, B - Genomic background of *Bt* clones tested by Rep-PCR, L - Ladder (1.5kb), C - PCR amplification of *cry3A* gene. Legend: 1 - *Bt* 407*cry* ^−^, 2 - *Bt* 407*gfpcry* ^−^, 3 - *Bt* 407*gfp-neocry* ^−^, 4- *Bt* 407*gfp-neocry* ^+^, 5-*Btt*, 6-*Btk*, L-ladder (1.0kb). Scale: 10 µm.
(TIF)

Table S1 **Insecticidal activity of different** ***Bt*** **strains to** ***T. castaneum*** **larvae.** Cox proportional hazard analysis testing the effect of treatment on survival (Figure S1). All bacteria strains were tested against the Naïve group. P-values less than 0.05 are shown in bold.
(DOC)

Table S2 **Dose response curves for** ***Btt*** **infection.** Cox proportional hazard analysis testing the effect of treatment on survival. All treatments were tested against Naïve group. P-values less than 0.05 are shown in bold.
(DOC)

Table S3 **Differences in susceptibility to** ***Btt*** **among ten beetle populations.** Cox proportional hazard analysis testing the effect of treatment on survival. All populations were tested against standard laboratory strain San Bernardino (SB). P-values less than 0.05 are shown in bold.
(DOC)

Table S4 **Plasmid exchange between** ***Btt*** **and the non-pathogenic** ***Bt*** **407** ***gfpcry*** ^−^ **– SB beetle population.** Cox proportional hazard analysis testing the effect of treatment on survival. All treatments were compared to *Bt* 407*gfp-neocry ^+^*. P-values less than 0.05 are shown in bold.
(DOC)

Table S5 **Plasmid exchange between** ***Btt*** **and the non-pathogenic** ***Bt*** **407** ***gfpcry*** ^−^ **– Cro1 beetle population.** Cox proportional hazard analysis testing the effect of treatment on survival. All treatments were compared to *Bt* 407*gfp-neocry ^+^*. P-values less than 0.05 are shown in bold.
(DOC)

Table S6 **Limited exposure time to** ***Btt*** **spore-containing diet.** Cox proportional hazard analysis testing the effect of treatment on survival. All treatment groups were compared to Naïve group. P-values less than 0.05 are shown in bold.
(DOC)

Table S7 **Larval mortality rate.** Cox proportional hazard analysis testing the effect of treatment on survival. All treatment groups were compared to *Bt* 407*gfp-neocry ^+^*. P-values less than 0.05 are shown in bold.
(DOC)
