# Correction: The Red Flour Beetle as a Model for Bacterial Oral Infections

**DOI:** 10.1371/journal.pone.0107599

**Published:** 2014-09-04

**Authors:** The PLOS ONE Staff

The third author’s name is spelled incorrectly. The correct name is: Robert Peuß. The correct citation is: Milutinović B, Stolpe C, Peuß R, Armitage SAO, Kurtz J (2013) The Red Flour Beetle as a Model for Bacterial Oral Infections. PLoS ONE 8(5): e64638. doi:10.1371/journal.pone.0064638
