# Supplementary figures and images for: The Red Flour Beetle as a Model for Bacterial Oral Infections
Source: PLoS One. 2013 May 30;8(5):e64638. doi: 10.1371/journal.pone.0064638 (PMC3667772; doi:10.1371/journal.pone.0064638)

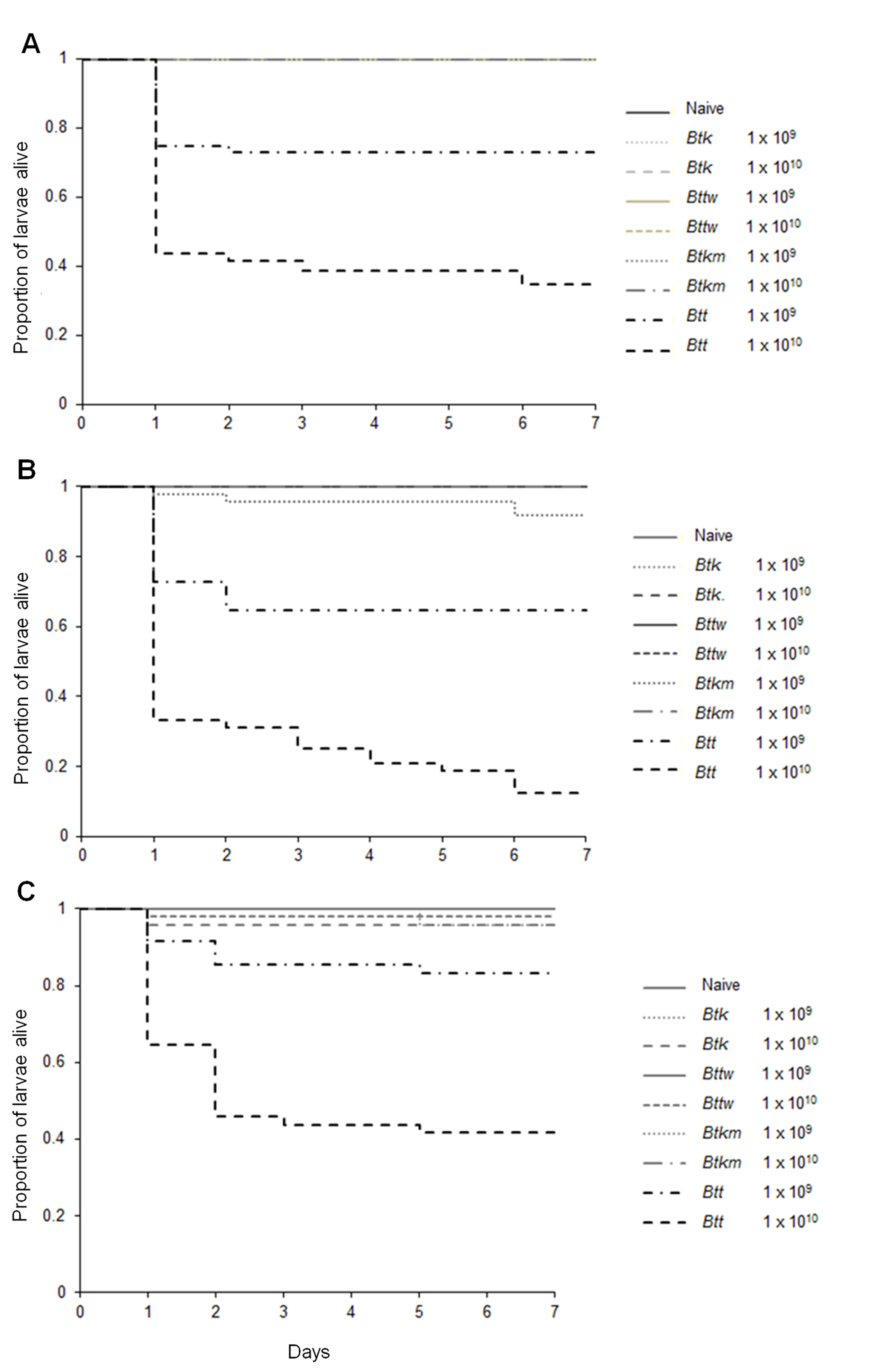

Supplement: Figure S1 — Insecticidal activity of different Bt strains to T. castaneum larvae - survival during the seven days of exposure. Larval survival during the seven days of constant exposure to flour containing Bt spores with two different concentrations, 109 mL−1 and 1010 mL−1 of four different Bt strains. Insect populations infected: A - San Bernardino (SB), B - Georgia 2 (GA-2), C - Croatia 1 (Cro1). (TIF) [file pone.0064638.s001.tif]

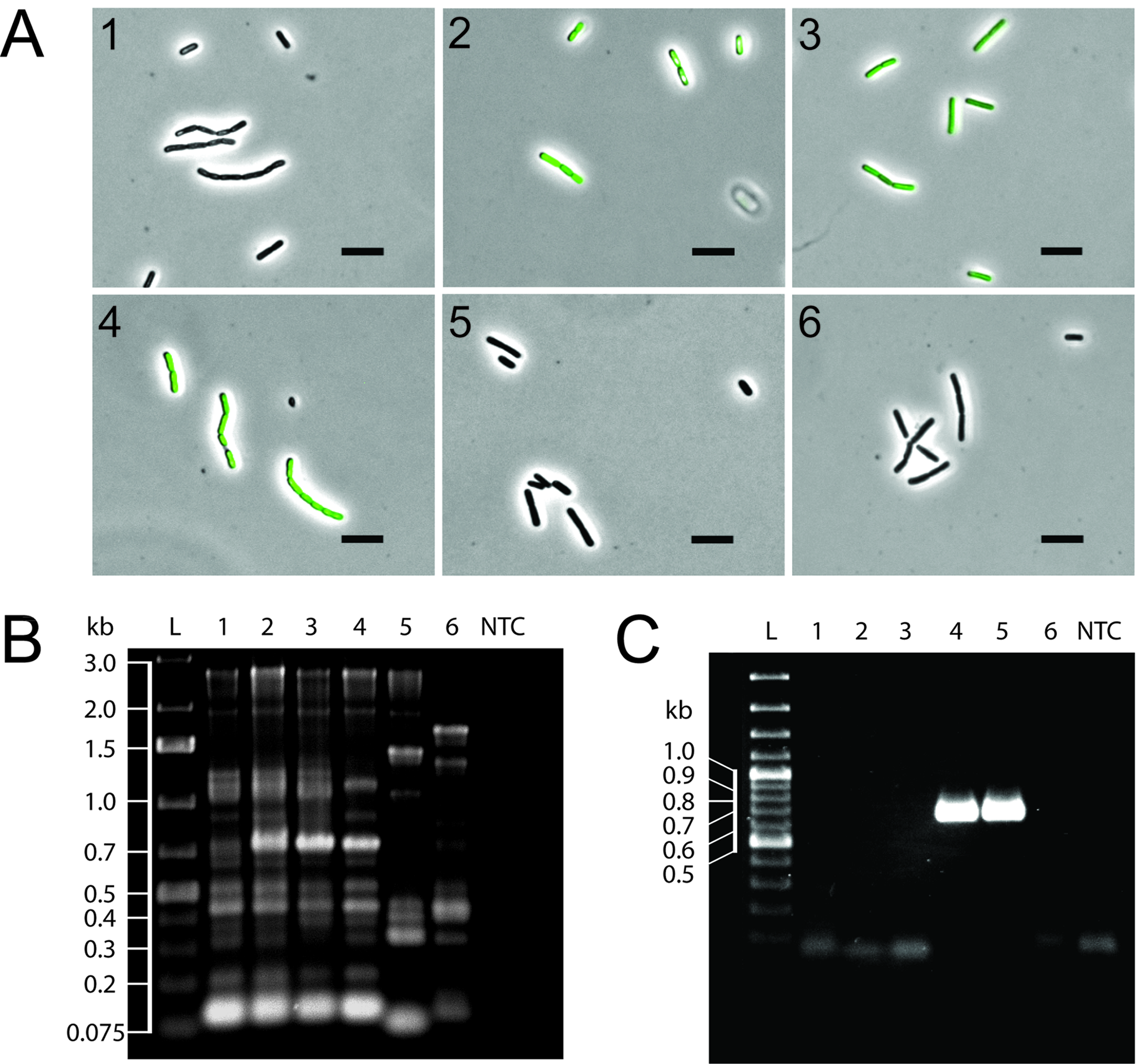

Supplement: Figure S2 — Characterisation of bacterial clones after the conjugation. A - Bt vegetative cells, phase contrast merged with fluorescence (GFP) microscopy, B - Genomic background of Bt clones tested by Rep-PCR, L - Ladder (1.5kb), C - PCR amplification of cry3A gene. Legend: 1 - Bt 407cry −, 2 - Bt 407gfpcry −, 3 - Bt 407gfp-neocry −, 4- Bt 407gfp-neocry +, 5-Btt, 6-Btk, L-ladder (1.0kb). Scale: 10 µm. (TIF) [file pone.0064638.s002.tif]
