# Supplementary material for: The Red Flour Beetle as a Model for Bacterial Oral Infections
Source: PLoS One. 2013 May 30;8(5):e64638. doi: 10.1371/journal.pone.0064638 (PMC3667772; doi:10.1371/journal.pone.0064638)
Supplement: Table S1 — Insecticidal activity of different Bt strains to T. castaneum larvae. Cox proportional hazard analysis testing the effect of treatment on survival (Figure S1). All bacteria strains were tested against the Naïve group. P-values less than 0.05 are shown in bold. (DOC) [file pone.0064638.s003.doc]

Table S1. Insecticidal activity of different *Bt* strains to *T. castaneum* larvae

|  | *Likelihood ratio* | *p* | *d.f.* | z | *p* |
| --- | --- | --- | --- | --- | --- |
| *n _total_ = 1296* |  |  |  |  |  |
| *Overall model* | *411.1* | ***<0.0001*** | *10* |  |  |
| *SB vs. Cro1* |  |  |  | *-0.937* | *0.349* |
| *SB vs. GA-2* |  |  |  | *2.484* | ***0.013*** |
| *Btt 1 x 10 ^9^* |  |  |  | *4.463* | ***<0.0001*** |
| *Btt 1 x 10 ^10^* |  |  |  | *6.870* | ***<0.0001*** |
| *Btk 1 x 10 ^9^* |  |  |  | *1.49^-13^* | *1* |
| *Btk 1 x 10 ^10^* |  |  |  | *0.375* | *0.707* |
| *Bttw 1 x 10 ^9^* |  |  |  | *1.38^-13^* | *1* |
| *Bttw 1 x 10 ^10^* |  |  |  | *-0.003* | *0.998* |
| *Btkm 1 x 10 ^9^* |  |  |  | *1.221* | *0.222* |
| *Btkm 1 x 10 ^10^* |  |  |  | *1.46^-13^* | *1* |
