# Supplementary material for: The Red Flour Beetle as a Model for Bacterial Oral Infections
Source: PLoS One. 2013 May 30;8(5):e64638. doi: 10.1371/journal.pone.0064638 (PMC3667772; doi:10.1371/journal.pone.0064638)
Supplement: Table S2 — Dose response curves for Btt infection. Cox proportional hazard analysis testing the effect of treatment on survival. All treatments were tested against Naïve group. P-values less than 0.05 are shown in bold. (DOC) [file pone.0064638.s004.doc]

Table S2. Dose response curves for *Btt* infection

| |  | *Likelihood ratio* | *p* | *d.f.* | z | *p* | | --- | --- | --- | --- | --- | --- | | *n _total_ =1584* |  |  |  |  |  | | *Overall model* | *936.2* | ***<0.0001*** | *12* |  |  | | *Cro1 vs. SB* |  |  |  | *-7.365* | ***<0.0001*** | | *GA-2 vs. SB* |  |  |  | *0.005* | *0.996* | | *1 x 10 ^6^* |  |  |  | *0.345* | *0.730* | | *1 x 10 ^7^* |  |  |  | *-0.378* | *0.705* | | *1 x 10 ^8^* |  |  |  | *0.330* | *0.741* | | *5 x 10 ^8^* |  |  |  | *3.643* | ***0.0003*** | | *1 x 10 ^9^* |  |  |  | *4.516* | ***<0.0001*** | | *3 x 10 ^9^* |  |  |  | *6.369* | ***<0.0001*** | | *5 x 10 ^9^* |  |  |  | *6.916* | ***<0.0001*** | | *7 x 10 ^9^* |  |  |  | *7.142* | ***<0.0001*** | | *1 x 10 ^10^* |  |  |  | *8.263* | ***<0.0001*** | | *5 x 10 ^10^* |  |  |  | *8.954* | ***<0.0001*** | |  |  |  |  |  |
| --- | --- | --- | --- | --- | --- | --- | --- | --- | --- | --- | --- | --- | --- | --- | --- | --- | --- | --- | --- | --- | --- | --- | --- | --- | --- | --- | --- | --- | --- | --- | --- | --- | --- | --- | --- | --- | --- | --- | --- | --- | --- | --- | --- | --- | --- | --- | --- | --- | --- | --- | --- | --- | --- | --- | --- | --- | --- | --- | --- | --- | --- | --- | --- | --- | --- | --- | --- | --- | --- | --- | --- | --- | --- | --- | --- | --- | --- | --- | --- | --- | --- | --- | --- | --- | --- | --- | --- | --- | --- | --- | --- | --- | --- | --- | --- |
