# Supplementary material for: The Red Flour Beetle as a Model for Bacterial Oral Infections
Source: PLoS One. 2013 May 30;8(5):e64638. doi: 10.1371/journal.pone.0064638 (PMC3667772; doi:10.1371/journal.pone.0064638)
Supplement: Table S3 — Differences in susceptibility to Btt among ten beetle populations. Cox proportional hazard analysis testing the effect of treatment on survival. All populations were tested against standard laboratory strain San Bernardino (SB). P-values less than 0.05 are shown in bold. (DOC) [file pone.0064638.s005.doc]

Table S3. Differences in susceptibility to *Btt* among ten beetle populations

|  | *Likelihood ratio* | *p* | *d.f.* | z | *p* |
| --- | --- | --- | --- | --- | --- |
| *n _total_ = 924* |  |  |  |  |  |
| *Overall model* | *76.46* | ***<0.0001*** | *10* |  |  |
| *Cro1* |  |  |  | *-2.527* | ***0.011*** |
| *Cro2* |  |  |  | *-5.696* | ***<0.0001*** |
| *GA-2* |  |  |  | *-0.815* | *0.856* |
| *OC* |  |  |  | *-2.918* | *0.051* |
| *43* |  |  |  | *0.177* | *0.055* |
| *50* |  |  |  | *-1.948* | ***<0.0001*** |
| *51* |  |  |  | *-1.922* | *0.415* |
| *57* |  |  |  | *-5.005* | ***0.003*** |
| *61* |  |  |  | *-3.004* | ***0.004*** |
