# Supplementary material for: The Red Flour Beetle as a Model for Bacterial Oral Infections
Source: PLoS One. 2013 May 30;8(5):e64638. doi: 10.1371/journal.pone.0064638 (PMC3667772; doi:10.1371/journal.pone.0064638)
Supplement: Table S4 — Plasmid exchange between Btt and the non-pathogenic Bt 407 gfpcry − – SB beetle population. Cox proportional hazard analysis testing the effect of treatment on survival. All treatments were compared to Bt 407gfp-neocry +. P-values less than 0.05 are shown in bold. (DOC) [file pone.0064638.s006.doc]

Table S4**.** Plasmid exchange between *Btt* and the non-pathogenic *Bt* 407*gfpcry* - – SB beetle population

| | |  | *Likelihood ratio* | *p* | *d.f.* | z | *p* | | --- | --- | --- | --- | --- | --- | | *n _total_ = 384* |  |  |  |  |  | | *Overall model* | *161.4* | ***<0.0001*** | *3* |  |  | | *Bt* 407*cry -* |  |  |  | *-5.073* | ***<0.0001*** | | *Bt* 407*gfpcry -* |  |  |  | *-5.081* | ***<0.0001*** | | *Btt* |  |  |  | *4.413* | ***<0.0001*** | |  |  |  |  |  | | --- | --- | --- | --- | --- | --- | --- | --- | --- | --- | --- | --- | --- | --- | --- | --- | --- | --- | --- | --- | --- | --- | --- | --- | --- | --- | --- | --- | --- | --- | --- | --- | --- | --- | --- | --- | --- | --- | --- | --- | --- | --- | |  |  |  |  |  |
| --- | --- | --- | --- | --- | --- | --- | --- | --- | --- | --- | --- | --- | --- | --- | --- | --- | --- | --- | --- | --- | --- | --- | --- | --- | --- | --- | --- | --- | --- | --- | --- | --- | --- | --- | --- | --- | --- | --- | --- | --- | --- | --- | --- | --- | --- | --- | --- |
