# Supplementary material for: The Red Flour Beetle as a Model for Bacterial Oral Infections
Source: PLoS One. 2013 May 30;8(5):e64638. doi: 10.1371/journal.pone.0064638 (PMC3667772; doi:10.1371/journal.pone.0064638)
Supplement: Table S5 — Plasmid exchange between Btt and the non-pathogenic Bt 407 gfpcry − – Cro1 beetle population. Cox proportional hazard analysis testing the effect of treatment on survival. All treatments were compared to Bt 407gfp-neocry +. P-values less than 0.05 are shown in bold. (DOC) [file pone.0064638.s007.doc]

Table S5. Plasmid exchange between *Btt* and the non-pathogenic *Bt* 407*gfpcry* - – Cro1 beetle population

| |  | *Likelihood ratio* | *p* | *d.f.* | z | *p* | | --- | --- | --- | --- | --- | --- | | *n _total_ = 384* |  |  |  |  |  | | *Overall model* | *105.9* | ***<0.0001*** | *3* |  |  | | *Bt* 407*cry –* |  |  |  | *-2.348* | ***0.019*** | | *Bt* 407*gfpcry –* |  |  |  | *-2.285* | ***0.022*** | | *Btt* |  |  |  | *5.153* | ***<0.0001*** | |  |  |  |  |  |
| --- | --- | --- | --- | --- | --- | --- | --- | --- | --- | --- | --- | --- | --- | --- | --- | --- | --- | --- | --- | --- | --- | --- | --- | --- | --- | --- | --- | --- | --- | --- | --- | --- | --- | --- | --- | --- | --- | --- | --- | --- | --- |
