# Supplementary material for: The Red Flour Beetle as a Model for Bacterial Oral Infections
Source: PLoS One. 2013 May 30;8(5):e64638. doi: 10.1371/journal.pone.0064638 (PMC3667772; doi:10.1371/journal.pone.0064638)
Supplement: Table S6 — Limited exposure time to Btt spore-containing diet. Cox proportional hazard analysis testing the effect of treatment on survival. All treatment groups were compared to Naïve group. P-values less than 0.05 are shown in bold. (DOC) [file pone.0064638.s008.doc]

Table S6. Limited exposure time to *Btt* spore-containing diet

|  | *Likelihood ratio* | *p* | *d.f.* | z | p |
| --- | --- | --- | --- | --- | --- |
| n total = 288 |  |  |  |  |  |
| Overall model | *55.91* | *<0.0001* | *5* |  |  |
| 30 min |  |  |  | 1.95-14 | 1 |
| 60 min |  |  |  | 0.981 | 0.326 |
| 90 min |  |  |  | 1.898 | 0.0577 |
| 120 min |  |  |  | 2.311 | **0.021** |
| 180 min |  |  |  | 3.427 | **0.001** |
