# Supplementary material for: The Red Flour Beetle as a Model for Bacterial Oral Infections
Source: PLoS One. 2013 May 30;8(5):e64638. doi: 10.1371/journal.pone.0064638 (PMC3667772; doi:10.1371/journal.pone.0064638)
Supplement: Table S7 — Larval mortality rate. Cox proportional hazard analysis testing the effect of treatment on survival. All treatment groups were compared to Bt 407gfp-neocry +. P-values less than 0.05 are shown in bold. (DOC) [file pone.0064638.s009.doc]

Table S7. Larval mortality rate

|  | *Likelihood ratio* | *p* | *d.f.* | z | *p* |
| --- | --- | --- | --- | --- | --- |
| *n _total_ = 480* |  |  |  |  |  |
| *Overall model* | *205.1* | ***<0.0001*** | *4* |  |  |
| *Naive* |  |  |  | *-1.683* | ***<0.0001*** |
| *Bt* 407*cry -* |  |  |  | *-2.349* | ***<0.0001*** |
| *Bt* 407*gfpcry –* |  |  |  | *-2.286* | ***<0.0001*** |
| *Btt* |  |  |  | *5.164* | ***0.007*** |
